# Diabetes Mellitus: A Risk Factor in Schlemm’s Canal-Based Minimally Invasive Glaucoma Surgery

**DOI:** 10.3390/jcm13247660

**Published:** 2024-12-16

**Authors:** Etsuo Chihara, Eri Nakano, Tomoyuki Chihara

**Affiliations:** 1 Sensho-kai Eye Institute, Minamiyama 50-1, Iseda, Kyoto 611-0043, Japan; mail4chiharat@gmail.com; 2 Department of Ophthalmology and Visual Sciences, Kyoto University Graduate School of Medicine, Kyoto 611-0043, Japan; nakaeri@kuhp.kyoto-u.ac.jp

**Keywords:** minimally invasive glaucoma surgery (MIGS), Schlemm’s canal-based surgery, diabetes mellitus, glaucoma, intraocular pressure, bleeding, surgical outcome, risk factor

## Abstract

**Objectives:** The objective of this study was to evaluate the impact of diabetes mellitus (DM) on the outcome of Schlemm’s canal-based minimally invasive glaucoma surgery (MIGS). **Methods**: In a retrospective interventional cohort study, postoperative intraocular pressure (IOP) and intracameral bleeding were analyzed in 25 diabetic patients and 84 non-diabetic patients, with primary open-angle glaucoma (POAG) or ocular hypertension (OH). **Results**: The mean follow-up period for all 109 eyes was 35.3 ± 24.8 months. There was no significant difference in preoperative IOP between cohorts with or without diabetes. However, the post-surgical IOP between 3 months and 2 years was significantly higher in the cohort with diabetes (*p* = 0.019 to 0.001). The 3-year survival probability of achieving an IOP ≤ 15 mmHg was 17.8 ± 0.09% in patients with diabetes, significantly lower than the 30.4 ± 0.06% observed in patients without diabetes (*p* = 0.042 Log-rank test). The 3-year survival probability of achieving an IOP ≤ 18 mmHg was 56.7 ± 0.12% in patients with diabetes compared to 79.5 ± 0.05% in patients without diabetes, indicating a marginally significant difference between cohorts with and without diabetes (*p* = 0.065). When the random effect of diabetes mellitus (DM) was analyzed alongside the fixed effects of preoperative IOP, age, refractive error, and the extent of canal opening using a multivariate linear mixed model, DM emerged as a significant risk factor for higher postoperative IOP at both 6 and 12 months (*p* < 0.001). **Conclusions**: Diabetes mellitus is a significant risk factor for poor outcomes following Schlemm’s canal-based MIGS, particularly in achieving lower postoperative IOP.

## 1. Introduction

Internal Schlemm’s canal opening surgery is a type of minimally invasive glaucoma surgery (MIGS) increasingly used to treat mild to moderate glaucoma.

Various devices, including Trabectome [1], Kahook dual blade (KDB) [2], Tanito microhook (TMH) [3], T-hook [4], and Gonioscopy-Assisted Transluminal Trabeculotomy (GATT) [5] have been developed and are now widely applied in clinical practice.

The surgical outcomes of these devices are generally similar, often resulting in postoperative intraocular pressures (IOPs) in the high teens [6].

Several factors, such as preoperative IOP, exfoliation glaucoma (EXG), IOP spikes, a history of selective laser trabeculoplasty or argon laser trabeculoplasty, younger age, high central corneal thickness, myopia, male gender, and Black ethnicity may influence postoperative IOP [7].

In addition to these factors, our clinical observations suggest that the reduction in IOP after surgery is poor in patients with diabetes.

To investigate the potential effects of diabetes mellitus (DM) on the surgical outcomes of Schlemm’s canal-based MIGS, we conducted a retrospective study on 232 patients who underwent combined cataract surgery and internal trabeculotomy at Sensho-kai Eye Institute.

## 2. Materials and Methods

Inclusion criteria were patients with cataracts and mild to moderate primary open-angle glaucoma (POAG) or ocular hypertension (OH) who were indicated for combined cataract surgery and minimally invasive Schlemm’s canal opening surgery (PI+SCO MIGS) due to poor visual acuity or inadequate IOP control despite the use of topical medications. Exclusion criteria included EXG, angle closure glaucoma (ACG), uveitic glaucoma, pigmentary glaucoma, and patients with poor compliance or incomplete data.

When the eye with glaucomatous optic neuropathy (GON: characterized by retinal nerve fiber layer defects and glaucomatous optic disk cupping) or glaucomatous retinal nerve fiber layer defects had a Shaffer grade of 2 or narrower angles on gonioscopy or an angle opening distance at 500 µm less than 0.2 mm as measured by anterior segment OCT (CASIA II, Tomey, Nagoya, Aichi, Japan), the eyes were diagnosed with chronic angle-closure glaucoma and excluded from this study. Between November 2016 and January 2024, 232 eyes underwent PI+SCO MIGS at Sensho-kai Eye Institute and were followed for more than 6 months. The surgeries included 81 eyes with KDB, 84 eyes with TMH, and 67 eyes with T-Hook. Among these, 24 eyes with EXG, 30 eyes with chronic ACG, 9 eyes with uveitic glaucoma, 1 eye with pigmentary glaucoma, and 10 eyes with poor compliance were excluded from this study. Of the remaining 158 eyes, 49 patients had bilateral surgeries, and data from their left eyes were excluded. Finally, 109 eyes of 109 patients were enrolled in this study.

Primary open-angle glaucoma (POAG) was defined as the presence of an open angle and GON, and glaucomatous abnormality detected using optical coherence tomography (OCT; AngioVue, RTVue XR; Optovue, Fremont, CA, USA). Visual field defects were evaluated with a Humphrey Field Analyzer (750i; Carl Zeiss Meditec, Tokyo, Japan). Ocular hypertension (OH) was defined as having an open angle and a history of high IOP exceeding 21 mmHg on at least two occasions, without glaucomatous visual field defects (normal glaucoma hemifield test and pattern standard deviation within 95% of normal on the Humphrey Field Analyzer) or glaucomatous optic neuropathy.

DM was diagnosed based on criteria established by the Japan Association for Diabetes Education and Care, defined as a fasting blood sugar level ≥ 126 mg/dL and/or HbA1C ≥ 6.5%.

The surgeries were performed by two of the authors (EC and TC). The surgical procedures have been reported previously [6]. Briefly, after the injection of viscoelastic material and anterior capsulorhexis, the internal trabeculotomy devices (KDB, TMH, and T-Hook) were inserted through a clear corneal opening at the 10 o’clock position, and the trabecular meshwork was incised over 120–150 degrees using a double-mirror Ahmed surgical goniolens (UADVX-H; Ocular, Bellevue, WA, USA). Following internal trabeculotomy, phacoemulsification, aspiration, and intraocular lens implantation were performed. After completion of cataract surgery, a 0.25% acetylcholine solution was injected into the anterior chamber (AC), and the corneal wound was closed with a single 10-0 nylon suture. Antiglaucoma medications were administered if postsurgical IOPs were elevated. Postoperatively, gatifloxacin, 0.1% betamethasone, and 2% pilocarpine eye drops were used 4 times per day for 2–4 weeks. Regarding the difference in surgical outcome between KDB, TMH, and T-Hook, we conducted a comparative study and found no difference in post-surgical IOP or gonioscopic findings [6].

Postsurgical bleeding into the anterior chamber in these patients was classified using the Shimane University grading system, which categorizes hyphema severity into 4 levels: L0, no hyphema; L1, layered bleeding <1 mm; L2 layered, bleeding ≥1 mm but not exceeding the pupillary margin; and L3, layered bleeding exceeding the inferior pupillary margin.

Blood clot (C) is categorized as C0, indicating no blood clots in the anterior chamber (AC), and 1 refers to the presence of blood clots. Floating red blood cells (R) are categorized as R0, indicating no floating RBCs in the AC; R1 refers to floating RBCs with clearly visible iris patterns in the entire AC; R2 refers to floating RBCs with partially obscured iris patterns; and R3 refers to dense floating RBCs with completely obscured iris patterns [8].

In this study, preoperative IOP was defined as the highest IOP recorded under medication within three months prior to surgery. In contrast, preoperative 3-mean IOP was defined as the average of three IOP measurements taken under medications immediately before surgery.

### Statistical Analysis

We used analysis of variance (ANOVA), Wilcoxon signed rank test, Chi-square test (Fisher’s exact test), Kaplan–Meier life table analysis, Haberman’s residual analysis, Dunnet’s test, two-way factorial analysis of variance, multivariate linear mixed model, and multiple regression all packaged in Bell Curve for Excel (Social Survey Research Information Co., Tokyo, Japan).

## 3. Results

**Figure 1:**
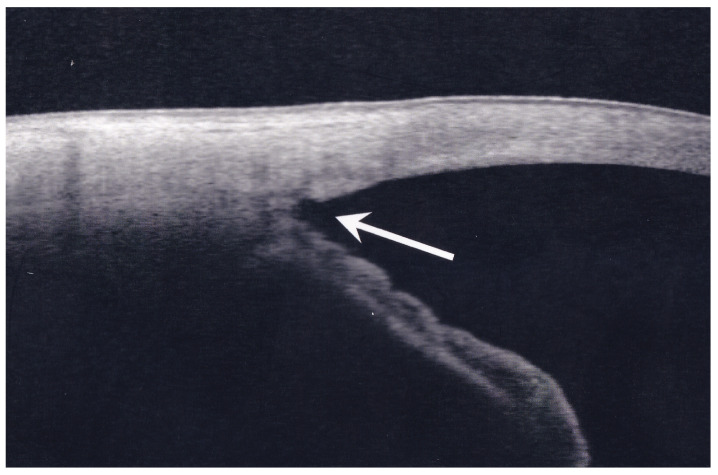
Anterior segment optical coherence tomography image of the angle following Kahook Dual Blade surgery. The arrow indicates the site of Schlemm’s canal opening.

The mean follow-up period was 35.3 ± 24.8 months (range 6.0–94.2 months). An example of an anterior segment optical coherence tomography image of the angle following internal canal opening surgery is presented in Figure 1.

Demographic data are presented in Table 1. There were no significant differences in baseline characteristics between the cohorts with or without diabetes, including pre-surgical IOP (highest IOP within 3 months prior to MIGS), logMAR best corrected visual acuity, refractive error, mean deviation on Humphrey visual field analysis, extent of canal opening, and follow-up duration (Table 1).

### 3.1. Postoperative Course of IOP and Medications After Canal Opening MIGS

Post-surgical IOP significantly decreased in both of the cohorts with and without diabetes as determined by the Wilcoxon signed-rank test (*p* < 0.01). However, the cohort with diabetes exhibited significantly higher post-surgical IOPs compared to the cohort without diabetes between three months (*p* = 0.001 by ANOVA) and two years (*p* = 0.019 by ANOVA) following surgery (Table 2).

To evaluate the overall effects of diabetes, to confirm differences in IOP between cohorts with and without diabetes at each time point, and to confirm time-dependent changes in IOP, a two-way factorial analysis of variance was performed. Here, again, a significant difference in IOP between subjects with and without diabetes was observed (Table 2). The *p*-values for the differences at each time point are provided in the 7th line of Table 2. The homoscedasticity of the data was confirmed using Bartlett’s test, which showed no significant deviation (*p* > 0.05). Using the Dunnet procedure, the overall difference between subjects with and without diabetes was found to be significant, with *p* = 0.0017 and F = 10.40 at 6 months and *p* = 0.0033 and F = 9.38 at 2 years.

Additionally, this analysis revealed significant differences between pre-surgical and post-surgical IOP at 3 months, 6 months, 1 year, 2 years, 3 years, and 4 years in both cohorts with and without diabetes, all with *p*-values < 0.001. The only exception was the comparison between pre-surgical IOP and IOP at 2 years in subjects with diabetes, where the *p*-value was 0.015.

Regarding post-surgical medications, the number of medications decreased significantly in both the DM (*p* = 0.03 to <0.001 for 3 years) and non-DM cohorts (*p* = 0.03 to <0.001 for 5 years; signed-rank test), with no significant difference observed between the two groups (Table 3).

Regarding sex-dependent variation in IOP, no significant differences were observed in preoperative IOP or postoperative IOP between male and female patients in either cohorts with or without diabetes (*p* > 0.05), except at 3 years postoperatively. At this point, female patients without diabetes had higher IOPs (16.3 ± 2.8 mmHg) compared to male patients without diabetes (13.9 ± 3.2 mmHg, *p* = 0.016). Similarly, female patients with diabetes had higher IOPs (18.3 ± 2.0 mmHg) compared to male patients without diabetes (14.7 ± 3.2 mmHg, *p* = 0.030).

**Figure 2:**
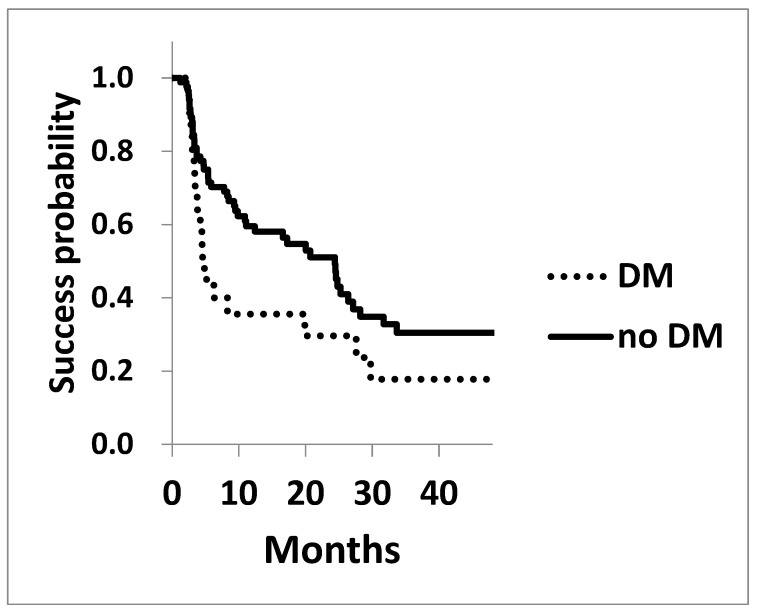
The success probability of achieving an IOP of 15 mmHg in patients with (dotted line) and without (solid line) diabetes. The survival probability of achieving a target of 15 mmHg, assessed using Kaplan–Meier life table analysis, was significantly lower in the cohort with diabetes (*p* = 0.042).

**Figure 3:**
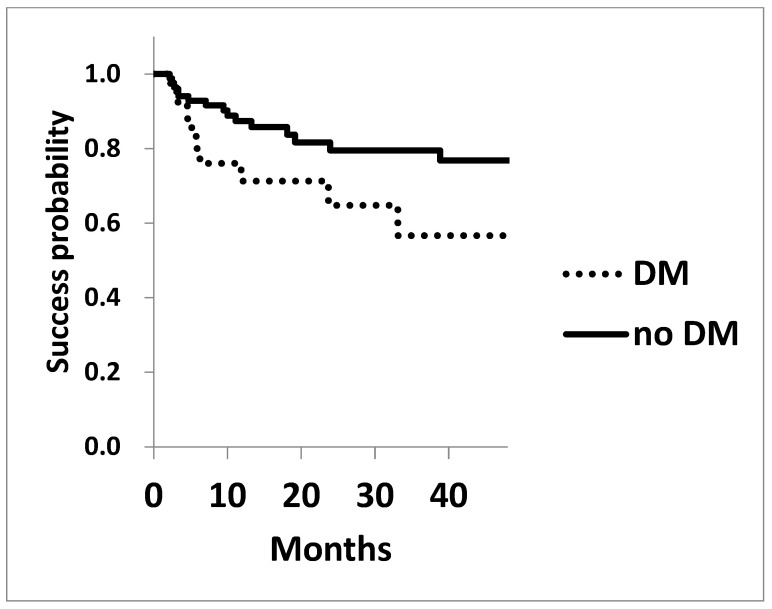
The success probability of achieving an IOP of 18 mmHg in patients with (dotted line) and without (solid line) diabetes. The survival probability of achieving a target IOP of 18 mmHg was assessed using Kaplan–Meier life table analysis. The success probability was marginally lower in the cohort with diabetes; *p* = 0.065.

The Kaplan–Meier survival analysis for achieving post-surgical IOPs of 15 mmHg and 18 mmHg is shown in Figure 2 and Figure 3. The survival probability of achieving 15 mmHg at three years in patients with diabetes was 17.8 ± 0.09%, significantly lower compared to that of the cohorts without diabetes of 30.4 ± 0.06% (*p* = 0.042 Log rank test, Figure 2). Similarly, the probability of achieving 18 mmHg at three years in cohorts with or without DM was 56.7 ± 0.12% and 79.5 ± 0.05% (*p* = 0.065), respectively, showing a trend toward worse outcomes in cohorts with diabetes (Figure 3).

### 3.2. Postoperative Intra-Cameral Bleedings

Regarding post-surgical intracameral bleeding, there was no significant difference in the prevalence of hyphema (L), clot formation (C), or bleeding density (R) between the groups (Table 4).

Regarding post-surgical intracameral bleeding and IOP spike, the prevalence of hyphema graded as L ≥ 2 hyphema and post-surgical IOP spike greater than 10 mmHg were significantly higher in male patients without diabetes compared to female patients without diabetes, with *p* = 0.020 (Table 5) and *p* = 0.040 (Table 6), respectively. However, this trend was not observed in patients with diabetes.

### 3.3. Results of Multivariate Analysis

The effects of DM on post-surgical IOP at 12 months were analyzed using a multivariate linear mixed model (Table 7). The fixed effects of variables such as age (*p* = 0.369), refractive error (*p* = 0.448), and extent of canal opening (*p* = 0.444) were not statistically significant, indicating no significant association between these three variables and post-surgical IOP. The fixed effects of pre-surgical IOP (*p* = 0.086) were marginal. However, the random effect of DM on 12-month post-surgical IOP was statistically significant (*p* < 0.001), with a standard deviation of 0.777, a variance component of 0.604, and a Chi-square value of 12.33 (Table 7), suggesting that DM contributes to variability in post-surgical IOP outcomes.

Similarly, when the effects of DM on post-surgical IOP at 6 months were assessed using the same model (Table 8), the fixed effects of age (*p* = 0.799), refractive error (*p* = 0.943), preoperative IOP (*p* = 0.119), and extent of canal opening (*p* = 0.954) were not statistically significant. However, the random effect of DM on post-surgical IOP at 6 months was statistically significant (*p* < 0.001), with a standard deviation of 0.787, a variance component of 0.619, and a Chi-square value of 11.06 (Table 8).

In another study, the associations between numerical variables and post-surgical IOP, excluding the effects of DM, were assessed using multiple regression analysis (Table 9 and Table 10).

In this case, preoperative IOP was significantly associated with post-surgical IOP at both 6 months (*p* < 0.001) and 12 months (*p* < 0.001). However, other parameters such as age, refractive error, and extent of canal opening showed no significant association with post-surgical IOP at either time point.

## 4. Discussion

To the best of the authors’ knowledge, no studies have specifically investigated the relationship between canal-based MIGS and diabetes. However, several studies on other types of glaucoma surgeries suggest that diabetes mellitus (DM) negatively impacts the outcomes of trabeculectomy and selective laser trabeculoplasty [9,10,11].

The underlying reasons for poorer surgical outcomes with diabetes remain unclear. The possibility that the extent of postoperative intraocular bleeding influences postoperative intraocular pressure is considered low because there were no observed differences in post-surgical hyphema or clot formation between patients with and without diabetes (Table 4).

Post-surgical wound healing in the angle after canal-opening surgery in human eyes has been extensively studied. Hamanaka and colleagues investigated the histopathology of the trabecular meshwork in eyes with failed canal-opening surgery. Their study found that Schlemm’s canal remained open in 10 of 31 eyes at six years post-surgery. In these cases, the inner wall of Schlemm’s canal was either covered or filled with fibrous tissue or regenerated endothelium of Schlemm’s canal. The structure of the trabecular meshwork was often lost and replaced by cell- and fiber-rich tissues [12,13].

While their study did include diabetic eyes, other studies have reported abnormalities associated with diabetes that may influence wound healing and outflow facility. Li et al. identified activation of Tyrosine kinase with immunoglobulin-like and EGF-like domains in diabetic eyes [14]. Ishikawa reported high expression of periostin and tenascin-C in the trabecular meshwork of patients with diabetes [15]. These abnormalities could potentially modulate wound healing and impair the outflow facility of the trabecular meshwork in diabetic eyes. If a target molecule responsible for these abnormalities is identified and a corresponding drug developed, innovative delivery methods such as nanocages—capable of delivering drugs at high concentrations to the target site—could become a practical treatment approach [16].

In patients with diabetes, post-surgical inflammation tends to be pronounced, while angiogenesis and fibroblast proliferation in the sclera and conjunctiva are reduced [17]. These factors, along with lower collagen synthesis and structural changes, may impair the ability of the tissue to contract and result in increased collagen density, granulation, and enhanced re-epithelialization [17]. Additionally, the breakdown of the blood–aqueous barrier, the release of cytokines, and persistent inflammation may alter the remodeling of surgical wounds, potentially contributing to poorer outcomes.

In the statistical analysis of the relationship between postoperative IOP and preoperative IOP, multiple regression revealed a significant correlation (*p* < 0.001) between preoperative IOP and IOP values at 6 months or 12 months post-surgery. However, in the multivariate linear mixed model, the relationship between preoperative IOP and IOP at 6 months post-surgery showed marginal significance with *p* = 0.08.

Since the purpose of the multivariate linear mixed model is to examine the random effect of DM, there is a possibility that confounding effects due to DM are involved. Additionally, the multivariate linear mixed model is considered to examine the relationship between preoperative IOP and the variability in post-surgical IOP outcomes, which may explain the discrepancy in results compared to the linear regression analysis.

In patients without diabetes, post-surgical hyphema and the prevalence of IOP spikes were significantly more common in males than in females. The reasons for this phenomenon remain unclear; however, this finding aligns with results from clinical studies suggesting that male gender is a risk factor for surgical failure [18].

## 5. Limitations

This study has several limitations. First, the sample size of subjects with diabetes is relatively small. Second, the retrospective design may introduce unforeseen biases that could affect the results. Lastly, the severity and duration of diabetes, which could potentially influence the outcome, were not assessed in this study and should be addressed in future research.

## 6. Conclusions

We analyzed surgical outcomes of concomitant Schlemm’s canal opening MIGS and cataract surgery in 25 patients with diabetes and 84 patients without who had POAG or ocular hypertension. Post-surgical IOP decreased significantly in both the cohorts with and without diabetes. However, the reduction in IOP was significantly smaller in the cohort with diabetes compared to the cohort without. These findings suggest that diabetes mellitus is a significant risk factor for achieving successful IOP control after Schlemm’s canal-opening minimally invasive glaucoma surgery.

## Figures and Tables

**Table 1:** Demographic data in cohorts with or without diabetes.

	Age	Sex F/M	Prev	Log MAR	Ref	MD	Pre-Op IOP	3 Mean Pre IOP	Extent	f/u Period
DM *n* = 25	71.6 ± 6.8	9/16	1	0.131 ± 0.263	−3.30 ± 4.19	−8.92 ± 7.11	22.0 ± 3.7	18.3 ± 2.9	130 ± 32	31.8 ± 24.2
No DM *n* = 84	69.2 ± 9.0	42/42	3	0.133 ± 0.304	−4.65 ± 4.77	−9.77 ± 8.32	20.9 ± 5.3	17.4 ± 3.4	145 ± 41	34.8 ± 24.9
*p*	0.215	0.258	1.00	0.975	0.211	0.668	0.345	0.248	0.118	0.604

Prev.: number of previous intraocular surgeries. LogMAR: Best corrected visual acuity using Logarithm of Minimum Angle Resolution. Ref: refractive error. MD: mean deviation using Humphrey visual field analyzer. Extent: the extent of canal opening. *p*: *p* by ANOVA or Fisher’s exact test.

**Table 2:** Course of intraocular pressure.

	Pre-Op	3 M	6 M	12 M	2 Y	3 Y	4 Y
DM	22.0 ± 3.7	16.8 ± 2.7	16.4 ± 3.0	16.1 ± 3.0	17.2 ± 3.5	16.6 ± 3.0	16.8 ± 2.6
*n*	25	25	25	22	17	13	10
No DM	20.9 ± 5.3	14.1 ± 3.6	14.3 ± 2.9	14.4 ± 3.0	14.9 ± 3.2	15.0 ± 3.2	14.7 ± 3.2
*n*	84	84	84	69	46	41	26
*p*+	0.345	0.001 *	0.003 *	0.022 *	0.019 *	0.126	0.089
*p*++	0.026 *	<0.001 *	<0.001 *	<0.001 *	<0.001 *	0.0014 *	<0.001 *

*p*+ by ANOVA, *p*++ by a two-way factorial analysis of variance. * Statistically significant by *p* < 0.05.

**Table 3:** Trends in the number of medications.

	Pre-Op	3M	12M	2Y	3Y	4Y
DM	2.6 ± 1.4	1.3 ± 1.0	1.5 ± 1.1	1.8 ± 1.1	1.9 ± 1.0	1.9 ± 0.9
*n*	25	25	22	17	13	10
No DM	2.9 ± 1.4	1.6 ± 1.3	1.6 ± 1.2	1.7 ± 1.4	1.7 ± 1.2	1.5 ± 1.0
*n*	84	84	69	46	41	26
*p*	0.335	0.203	0.711	0.957	0.540	0.280

**Table 4:** Haberman’s residual analysis of hyphema, clot formation and bleeding density.

	L0	L1	L2	L3	C0	C1	R0	R1	R2	R3
no DM = 84	49	14	18	3	58	26	17	43	15	9
DM = 25	16	3	5	1	19	6	3	13	7	2
Two-tailed *p* using adjusted residual	0.719	0.594	0.887	0.921	0.678	0.553	0.384	0.957	0.305	0.703

**Table 5:** Prevalence of intracameral hyphema graded as L ≥ 2 in the cohort without diabetes.

L ≥ 2	Yes	No
Female	5	37
Male	15	27

**Table 6:** Prevalence of post-surgical IOP spikes ≥ 10 mmHg in the cohort without diabetes.

Spike ≥ 10	Yes	No
Female	10	32
Male	20	22

**Table 7:** Fixed effects of ocular variables on IOP at 12 months: a linear mixed model analysis.

			95% Confidence Interval of Partial Regression Coefficient			
Variable	Partial Regression Coefficient	Standard Error	Lower Limit	Upper Limit	*t* Value	*p* Value
age	−0.055	0.036	−0.512	0.402	−1.538	0.3689
refractive error	−0.08	0.068	−0.94	0.781	−1.177	0.4483
pre-op IOP	0.514	0.07	−0.378	1.407	7.326	0.0864
extent of excision	−0.007	0.06	−0.079	0.066	−1.193	0.4441
constant term	15.27	0.613	7.48	23.066	24.9	0.0256

**Table 8:** Fixed effects of ocular variables on IOP at 6 months: A linear mixed model analysis.

			95% Confidence Interval of Partial Regression Coefficient			
Variable	Partial Regression Coefficient	Standard Error	Lower Limit	Upper Limit	*t* Value	*p* Value
age	−0.013	0.039	−0.506	0.481	−0.327	0.799
refractive error	−0.06	0.069	−0.881	0.869	−0.089	0.944
pre-op IOP	0.415	0.078	−0.583	1.413	5.281	0.119
extent of excision	0.001	0.006	−0.081	0.072	0.072	0.954
constant term	15.23	0.63	7.226	24.172	24.172	0.026

**Table 9:** Association between 12-month post-surgical IOP and four dependent variables, analyzed using multiple regression.

			95% Confidence Interval of Partial Regression Coefficient			
Variables	Partial Regression Coefficient	Standard Error	Lower Limit	Upper Limit	*t* Value	*p* Value
age	−0.047	0.039	−0.123	0.029	−1.223	0.225
refractive error	−0.066	0.072	−0.21	0.078	−0.914	0.364
pre-op IOP	0.532	0.075	0.383	0.681	7.092	<0.001
extent of excision	−0.009	0.006	−0.021	0.003	−1.507	0.136
constant term	9.73	3.392	2.981	16.47	2.868	0.005

**Table 10:** Association between 6-month post-surgical IOP and four dependent variables, analyzed using multiple regression.

			95% Confidence Interval of Partial Regression Coefficient			
Variables	Partial Regression Coefficient	Standard Error	Lower Limit	Upper Limit	*t* Value	*p* Value
age	−0.006	0.041	−0.087	0.075	−0.142	0.887
refractive error	0.001	0.073	−0.143	0.145	0.012	0.990
pre-op IOP	0.438	0.082	0.275	0.601	5.327	<0.001
extent of excision	−0.002	0.007	−0.015	0.012	−0.264	0.792
constant term	7.776	3.72	0.398	15.154	2.091	0.039

## Data Availability

The research data used in this research are available upon request from the corresponding author.
